# Optimal patient selection for maze procedure in patients undergoing mitral valve disease

**DOI:** 10.1186/s13019-024-02766-z

**Published:** 2024-04-08

**Authors:** Tomoaki Masuda, Atsushi Aoki, Tadashi Omoto, Kazuto Maruta

**Affiliations:** Department of Cardiovascular Surgery, Showa University, Hatanodai 1-5-8, Shinagawa-Ku, Tokyo, 142-8666 Japan

**Keywords:** MAZE, Mitral Valve Surgery, Atrial Fibrillation, Predictors

## Abstract

**Objectives:**

Although risk factors for unsuccessful Maze procedure have been demonstrated, an appropriate patient selection is still controversial. In our institute, Maze procedure is indicated for those whom normal sinus rhythm (NSR) was reestablished by intraoperative direct cardioversion (DC) after ventricular unloading by total cardiopulmonary bypass. The purpose of this study was to evaluate the effectiveness of our indication criteria for Maze procedure in patients with mitral valve disease.

**Methods:**

Between October 2012 and October 2021, MAZE was indicated in 55 patients in whom normal sinus rhythm (NSR) was reestablished by intraoperative direct current cardioversion (DC). Three endpoints and predictors were examined: disappearance of atrial fibrillation (AF), NSR, and A-wave detection.

**Results:**

Restoration of NSR by intraoperative DC was confirmed in 43 patients, and these patients underwent MAZE. AF disappeared in 39 patients (90.7%), and F-wave ≥ 0.1 mV was a significant predictive factor (odds ratio (OR) 20.99, 95% CI 1.22–1079.06). NSR was reestablished in 36 patients (83.7%), and F-wave ≥ 0.1 mV (odds ratio 15.62, 95% CI 1.62–359.86) + AF history ≤ 3 years (OR 8.30, 95% CI 1.09–177.04) were significant predictors. A-wave detection was confirmed in 26 patients (60.5%), and left atrial diameter ≤ 55 mm was a significant predictor (OR 5.22, 95% CI 1.28–24.79).

**Conclusions:**

Intraoperative DC after ventricular unloading resulted effective patient selection for concomitant Maze procedure. F-wave and AF history were predictive factor of electrical restoration of AF, and left atrial diameter was predictive factor of restoration of atrial function.

## Introduction

Atrial fibrillation (AF), a common finding in valvular heart disease, is associated with thromboembolic complications, and high surgical and mid- to long-term mortality [1, 2]. Particularly in patients with mitral valve disease, the frequency of persistent AF is reported to be 40% or more [3, 4]. It has been reported that an additional concomitant Maze procedure (MAZE) in mitral valve surgery does not increase surgical mortality, and reduces the risk of thromboembolism [5, 6].

However, the patient selection for concomitant Maze procedure is difficult, especially elder or high risk patients, because electrical or functional recovery might not always be achieved. Previous studies have demonstrated that F wave amplitude, AF period and left atrial diameter (LAD) are predictive factors for AF disappearance or recurrence after MAZE [7]. Recovery of atrial contractility is also an important clinical advantage by the Maze procedure. Previous studies have demonstrated that NSR is restored in 90% of cases, however, the frequency of atrial contractility is restored in 60% [8, 9].

We have performed Maze procedure in cases who showed restored NSR by DC after ventricular unloading after total cardiopulmonary bypass. The purpose of this study was to evaluate the effectiveness of our indication criteria for Maze procedure in patients with mitral valve disease.

## Methods

**Fig. 1:**
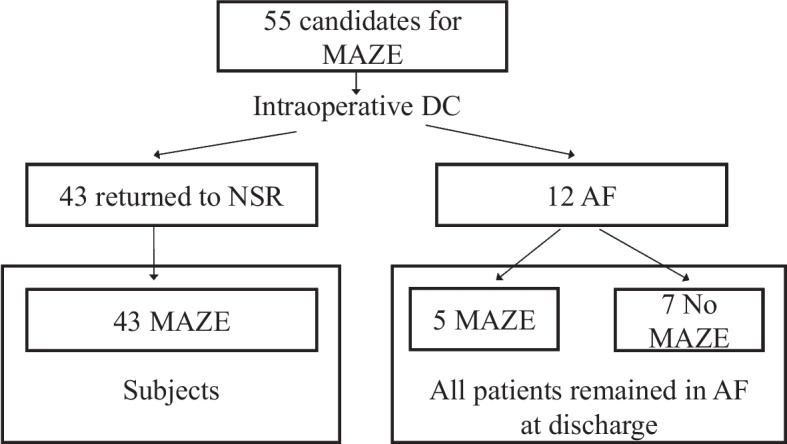
Subjects. Atrial de-fibrillation and sinus node function were evaluated by direct current cardioversion (DC) under complete left atrium unloading with total cardiopulmonary bypass. The Cox-maze IV procedure (MAZE) was then performed. AF, atrial fibrillation; NSR, normal sinus rhythm

The study complied with the Declaration of Helsinki, and was approved by the Institutional Review Board of Showa University School of Medicine (IRB No. 3123, approved on 2020.5.7). Patients were given opt-out information regarding the study. Between October 2012 and October 2021, 221 patients underwent mitral valve surgery. Persistent AF was documented in 81 patients pre-operatively. In these patients, MAZE was not indicated in 26 patients for the following reasons: previous cardiac surgery (*n* = 6), permanent AF (*n* = 14), high operative risk (*n* = 4), low ejection fraction (*n* = 1), and severe mitral annular calcification (*n* = 1). In our institution, we routinely perform direct current cardioversion (DC) under complete left atrial and ventricular unloading with total cardiopulmonary bypass, and MAZE is indicated in those in whom NSR was restored by DC. After total bypass was reached in well-decompressed hearts, the defibrillator paddles were positioned on both atria to the extent possible, and the heart was defibrillated with a synchronized shock of 30 J. Three successive failures to return to sinus rhythm was considered to indicate non-defibrillation. Of these 55 cases with mitral valve surgery, 43 cases whose cardiac rhythm returned to NSR by intraoperative DC were included in this study (Fig. 1). Five patients with medically uncontrollable AF tachycardia, who were classified as “non-defibrillation” have, nevertheless, underwent Maze procedure, because the clinical advantages AF disappearance were supposed to be significant. Those five patients were excluded from this study.

**Fig. 2:**
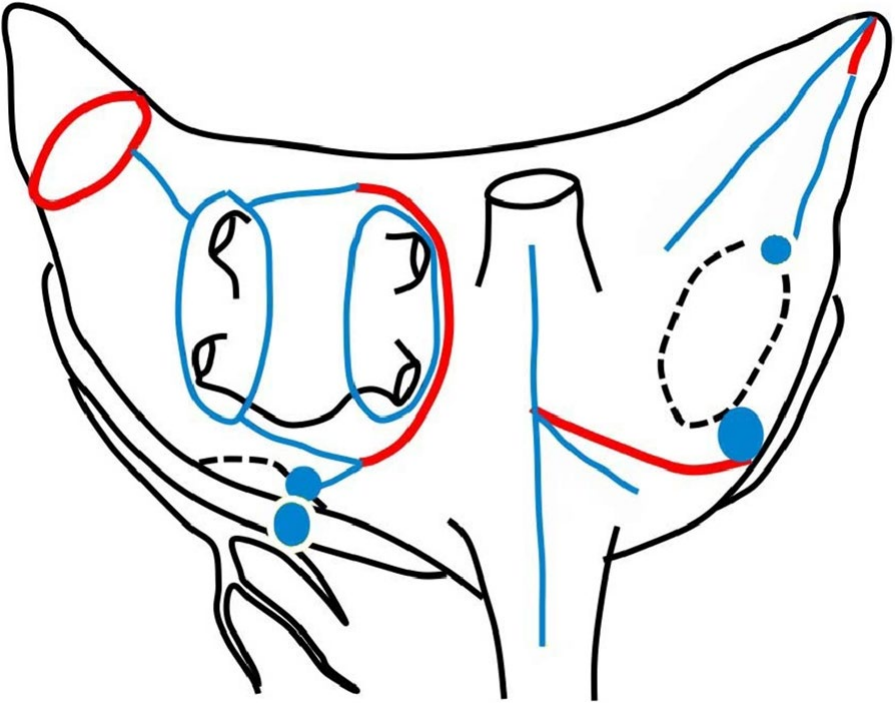
MAZE procedure. Red line: incision line. Blue line: ablation line

Our surgical strategy of Cox-maze IV procedure is according to the previous paper from Damiano et al. [10]. MAZE was conducted using the Cox-maze IV procedure via a bipolar and monopolar radiofrequency ablation system in all patients. In the right atrium, incision of the free wall, and ablation of the tricuspid annulus, right atrial appendage to the tricuspid annulus, ostium of the superior and inferior vena cava, and atrial septum were performed. In the left atrium, pulmonary vein isolation from the epicardial side and left atrial appendage resection, and box isolation were performed, and ablation to the mitral annulus was performed from the epicardial side via the coronary sinus. The left atrial appendage was surgically resected and closed by over-sewing with felt (Fig. 2). We routinely implant a temporary pacemaker wire on both the right atrial and right ventricular free wall, which is removed within 5–7 days postoperatively. Electrocardiogram was performed daily until the day of discharge.

F waves were measured in lead V1. For electrocardiogram recording, the sampling interval was 8 kHz, and digital filters (100 Hz EMG filter and 0.5 Hz baseline sway removal filter) were used. A 12-lead electrocardiogram with a regular rhythm and P waves in lead II or V1 was considered to indicate NSR.

If AF developed after surgery, DC was performed when palpitations and hypotension were observed. Otherwise, magnesium sulfate or pilsicainide hydrochloride hydrate was administered, and DC was performed if AF persisted for more than 24 h. If AF persisted even after fluid balance was stabilized, amiodarone was administered. Postoperative β-blockers were used if tachycardia persisted. The presence of the mitral A-wave was evaluated by transthoracic echocardiogram performed 7–10 days after surgery.

Three endpoints and clinical predictors were examined by multivariate analysis, namely (1) AF disappearance at discharge, (2) P-wave detection on ECG as electrical restoration of atrial function at discharge, and (3) A-wave detection on postoperative echocardiography to indicate mechanical restoration of atrial function. Clinical predictors were F-wave ≥ 0.1 mV, AF history ≤ 3 years, and LAD ≤ 55 mm. The demographic and clinical data were expressed as mean ± standard deviation or number (%). All analyses were performed using JMP Pro 15 (SAS Institute Inc., SAS Campus Drive, Cary, NC, USA), and a *p*-value < 0.05 was considered to indicate statistical significance.

## Results

Patient characteristics are shown in Table 1. Average age was 70.7 ± 8.2 years and 18 patients (41.9%) were female. Average AF duration was 47.4 ± 66.5 months, ranging from 2 months to 30 years, and 18 (42.9%) patients suffered AF longer than 3 years. Average F wave was 0.19 ± 0.09 mV, and 36 (83.7%) patients had an F wave above 0.1 mV. Average LAD was 51.2 ± 8.2 mm, and was 55 mm or more in 13 (30.2%) patients (Table 1). Primary mitral valve regurgitation (MR) was seen in 55.8% of patients, mostly due to prolapse (95.8%), and was treated with mitral valve plasty (MVP). Secondary MR was 27.9%, which had an etiology of AF in 66.7%, aortic valve disease in 16.7%, and cardiomyopathy in 16.7%. Rheumatic mitral valve stenosis (MS) occurred in 16.3% of patients, all of whom underwent mitral valve replacement (MVR) (Table 2). The average surgical procedure time was broken down as follows: operation time 287.5 ± 44.7 min, cardiopulmonary bypass time 161.0 ± 29.4 min, and the aortic cross clamp time 114.4 ± 22.9 min. Only 9.3% patients underwent isolated mitral valve surgery whereas 90.7% underwent combined valve surgery (Table 1). In 22 (51.2%) cases, postoperative atrial pacing at a set rate ≥ 80 bpm was performed for 3 ± 1.8 days (1–7 days). Postoperative brain natriuretic peptide (BNP) was 148.1 ± 166.8 (38.6–924.4) pg/ml, which was significantly decreased compared with a preoperative BNP of 297.0 ± 262.9 (35.7–1336.3) pg/ml. With regard to postoperative anticoagulation, warfarin was administered to all patients after mitral valve surgery. 16 (37.2%) cases received a postoperative beta-blocker, and 37 (86.0%) cases received a postoperative antiarrhythmic drug.

**Table 1:** Patinent profile and operation

	(***n*** = 43)
Age	70.7 ± 8.2
Sex (Female)	18 (41.9%)
Body height (cm)	162.0 ± 9.9
Body weight (kg)	58.1 ± 12.1
AF period (months)	47.4 ± 66.5 (2〜360)
AF period ≥ 3 years	18 (42.9%)
F-wave height (mV)	0.19 ± 0.09
F-wave ≥ 0.1 mV	36 (83.7%)
LAD (mm)	51.2 ± 8.2
LAD ≥ 55 mm	13 (30.2%)
EF (%)	54.2 ± 10.5
TRPG (mmHg)	33.0 ± 13.1
BNP (pg/ml)	297.0 ± 262.9
Preoperative oral beta blocker	30 (69.8%)
Preoperative oral antiarrhythmic drugs	11 (25.6%)
Operation time (min)	287.5 ± 44.7
Cardiopulmonary bypass time (min)	161.0 ± 29.4
Aortic cross clamp time (min)	114.4 ± 22.9
Isolated mitral valve surgery	4 (9.3%)
Combined valve surgery	39 (90.7%)
Simultaneous coronary artery bypass grafting	1 (2.3%)
Aortic valve surgery	0

*AF* atrial fibrillation, *LAD* left atrial diameter, *EF* ejection fraction, *TRPG* tricuspid regurgitation peak gradient, *BNP* brain natriuretic peptide

In 39 of 43 patients (90.7%), AF had disappeared at discharge, for which F-wave ≥ 0.1 mV was a significant predictive factor (odds ratio (OR) 20.99, 95% confidence interval (CI) 1.22–1079.06, *p* = 0.036). Electrical restoration of atrial function was observed in 36 patients (83.7%), for which F-wave ≥ 0.1 mV (odds ratio 15.62, 95% CI 1.62–359.86, *p* = 0.017) + AF history ≤ 3 years (OR 8.30, 95% CI 1.09–177.04, *p* = 0.041) were significant predictors. Mechanical atrial function restoration was confirmed in 26 patients (60.5%), and LAD ≤ 55 mm was a significant predictor (OR 5.22, 95% CI 1.28–24.79, *p* = 0.021) (Tables 3 and 4).

**Table 2:** Etiology and Procedure

	*n*=43	Etiology		Procedure	
Primary MR	24 (55.8%)	Prolapse	23 (95.8%)	MVP	23 (95.8%)
		Rheumatic	1 (4.2%)	MVR	1 (4.2%)
Secondary MR	12 (27.9%)	AF	8 (66.7%)	MAP	7 (58.3%)
		Aortic valve disease	2 (16.7%)	MVP	1 (8.3%)
		Cardiomyopathy	2 (16.7%)	MVR	4 (33.3%)
MS	7 (16.3%)	Rheumatic	7 (100%)	MVR	7 (100%)

*MR* mitral valve regurgitation, *MS* mitral valve stenosis, *AF* atrial fibrillation, *MVP* mitral valve plasty, *MAP* mitral annuloplasty, *MVR* mitral valve replacement

**Table 3:** Rhythm at discharge

	(*n* = 43)
AF disappearance at discharge	39 (90.7%)
Rhythm at discharge	
NSR	36 (83.7%)
AF	4 (9.3%)
Junctional rhythm	3 (7.0%)
NSR with A-wave	26 (60.5%)

*AF* atrial fibrillation, *NSR* normal sinus rhythm

In 43 patients, restoration of NSR was confirmed by intraoperative DC, and these 43 patients underwent MAZE. In 39 of 43 patients (90.7%), AF had disappeared by discharge. Electrical restoration of atrial function was observed in 36 patients (83.7%). Mechanical atrial function restoration was confirmed in 26 patients (60.5%). Two of those classified with postoperative AF underwent permanent PM implantation due to AF bradycardia

**Table 4:** Predictors

	Odds ratio	95% CI	*p* value
(1) The predictors of AF disappearance			
F-wave ≥ 0.1 mV	20.99	1.22 – 1079.06	0.036
AF duration ≤ 3 years	7.36	0.36 – 149.84	0.147
LAD ≤ 55 mm	9.90	0.57 – 172.65	0.079
(2) The predictors of NSR			
F-wave ≥ 0.1 mV	15.62	1.62 – 359.86	0.017
AF duration ≤ 3 years	8.30	1.09 – 177.04	0.041
LAD ≤ 55 mm	1.40	0.20 – 9.71	0.737
(3) The predictors of NSR with A-wave			
F-wave ≥ 0.1 mV	1.68	0.25 – 11.41	0.595
AF duration ≤ 3 years	1.96	0.49 – 7.82	0.342
LAD ≤ 55 mm	5.22	1.28 – 24.79	0.021

(1) Predictors of AF disappearance. The significant predictive factor of AF disappearance was F-wave ≥ 0.1 mV

(2) Predictors of NSR. Significant predictive factors of electrical restoration of AF were F-wave ≥ 0.1 mV + AF history ≤ 3 years

(3) Predictors of NSR with A-wave. The significant predictive factor of mechanical restoration of atrial function was a left atrial diameter ≤ 55 mm. 95% CI, 95% confidence interval; AF, atrial fibrillation; LAD, left atrial diameter

One patient died due to pneumonia 14 days after surgery. No other deaths were seen by 30 days. There were no cerebrovascular events during hospitalization. Two patients classified as having postoperative AF underwent permanent pacemaker implantation due to AF bradycardia.

## Discussion

In our study of patients undergoing mitral valve surgery with the Maze procedure, AF disappeared in 90.7% patients, NSR was reestablished in 83.7% patients, and A-wave detection was confirmed in 60.5% patients. Although our strategy for patient selection seemed to be successful, electrical or functional recovery has not been satisfactory. Three factors were predictive of these three endpoints: F-wave was predictive of AF disappearance; F-wave + AF history was predictive of electrical restoration of AF; and left atrial diameter was predictive of restoration of atrial function. These findings clarify which patients are most likely to benefit from the Maze procedure.

Cox and colleagues expected that atrial contraction would be maintained when AF returns to NSR. They also expected that a return of AF to NSR would decrease the risk of thromboembolism without anticoagulant therapy, cardiac output would increase, and survival rate would be improved, and accordingly devised MAZE for AF [11, 12]. A high NSR recovery rate (98%) for isolated AF was reported for the original Cox maze procedure [13]. Combined MAZE for AF during mitral valve surgery did not increase surgical mortality and had the merit of reducing the risk of thromboembolism and the requirement of long-term anticoagulant therapy [5, 6]. Further, long-term improvement in quality of life has been shown in patients on NSR maintenance after MAZE [14–16].

However, Feinberg et al*.* reported that only 61% of patients experienced left atrial contraction on echocardiography after MAZE, and their left atrial contractility was only about half that of normal subjects [8]. Further, Isobe et al*.* reported that although sinus rhythm was resumed in 90% of patients after MAZE, the frequency of atrial contraction detected by echocardiography was 66.7% [9]. Therefore, both electrical and functional atrial recovery is the optimal goal of the Maze procedure. The recovery of left atrial contraction is considered to be the greatest benefit of MAZE, or in other words the restoration of NSR followed by AF disappearance. Previously proposed risk factors for AF recurrence after MAZE, i.e., small F wave, long history of atrial fibrillation and giant left atrium, should be reassessed for each of the three endpoints (AF disappearance, restoration of NSR and recovery of atrial contraction).

Previous studies of mitral valve surgery combined with MAZE for AF have demonstrated risk factor of postoperative AF recurrence as left atrial size, duration of AF and F-wave voltage [7, 17]. However, the treatment advantages were rather focused only on recovery to NSR. However, we think that our three study endpoints are the treatment benefits of maze procedure: AF disappearance, electrical recovery and atrial functional recovery. In this stand point, we believe we have novelty in our study.

In our study, both restoration of NSR and recovery of atrial contraction were found in patients with a left atrial diameter of 55 mm or less. This result is considered to be associated with the weakening of atrial tissue in over-distended left atrium. A previous study demonstrated that the recovery of atrial contraction recovery was lower in patients with a giant left atrium than in those without, and that the left atrium does not contract when dilated even if cardiac rhythm returns to NSR [18].

A small F wave is associated with atrial fibrosis [19]. The possibility of AF disappearance in these cases is considered low; moreover, sinus node function might be impaired when the history of AF is long. Taking these findings into consideration, our finding that a large F wave (≥ 0.1 mV) is predictive of electrical and functional atrial recovery appears reasonable. Therefore, regarding indications for MAZE, even if MAZE is performed in patients with a large left atrium and NSR is reestablished, difficulty in restoring atrial contraction and the risk of embolic event remains. Accordingly, warfarin cannot be discontinued, and thus the benefit of MAZE in these patients may by small.

Intraoperative DC under complete left atrial and ventricular unloading by total cardiopulmonary bypass is a useful and effective method for selecting candidates for MAZE. Although few studies have investigated intraoperative DC before MAZE, several reports demonstrated that AF disappearance by DC before catheter ablation was a useful predictor of AF recurrence after catheter intervention [20–22]. Of our 12 patients who did not recover from intraoperative DC, we performed MAZE for 5 patients, even though NSR could not be restored by intraoperative DC because preoperative episodes of AF tachycardia were medically uncontrollable, and AF did not disappear in all patients. In addition, 7 patients who did not undergo MAZE remained in AF postoperatively. On the other hand, in patients with NSR restoration by intraoperative DC, AF was resolved in 90%. Further studies are warranted to confirm the usefulness of intraoperative DC as an indicator of successful MAZE.

Several limitations of our study warrant mention. First, the study was conducted at a single institution, and the ability to draw definitive conclusions is limited due to its small sample size and retrospective nature. Second, we do not report long-term follow-up data, and this is a subject for future study. Nevertheless, our study clarified which patients are most likely to benefit from the Maze procedure.

## Conclusions

In patients undergoing mitral valve surgery with Maze procedure, an F-wave ≥ 0.1 mV and F-wave ≥ 0.1 mV + AF history were predictive for AF disappearance and electrical restoration of AF, respectively. Left atrial diameter ≤ 55 mm was a predictive factor for functional restoration of atrial function, which is considered to be an important surgical benefit. Effective patient selection for concomitant maze procedure might be achieved by considering these three factors and intraoperative DC under ventricular unloading.

## Data Availability

Not applicable.

## Abbreviations

AF: Atrial fibrillation
MAZE: Maze procedure
LAD: Left atrial diameter
NSR: Normal sinus rhythm
DC: Direct current cardioversion
MR: Mitral valve regurgitation
MVP: Mitral valve plasty
MS: Mitral valve stenosis
MVR: Mitral valve replacement
BNP: Brain natriuretic peptide
OR: Odds ratio
CI: Confidence interval
